# A Non-woven Path: Electrospun Poly(lactic acid) Scaffolds for Kidney Tissue Engineering

**DOI:** 10.1007/s13770-017-0107-5

**Published:** 2018-02-14

**Authors:** Todd P. Burton, Anthony Callanan

**Affiliations:** Institute of Bioengineering, School of Engineering, The University of Edinburgh, Faraday Building, The King’s Buildings, Mayfield Road, Edinburgh, EH9 3JL UK

**Keywords:** Kidney tissue engineering, Scaffold architecture, Electrospinning, Primary cells, Renal

## Abstract

Chronic kidney disease is a major global health problem affecting millions of people; kidney tissue engineering provides an opportunity to better understand this disease, and has the capacity to provide a cure. Two-dimensional cell culture and decellularised tissue have been the main focus of this research thus far, but despite promising results these methods are not without their shortcomings. Polymer fabrication techniques such as electrospinning have the potential to provide a non-woven path for kidney tissue engineering. In this experiment we isolated rat primary kidney cells which were seeded on electrospun poly(lactic acid) scaffolds. Our results showed that the scaffolds were capable of sustaining a multi-population of kidney cells, determined by the presence of: aquaporin-1 (proximal tubules), aquaporin-2 (collecting ducts), synaptopodin (glomerular epithelia) and von Willebrand factor (glomerular endothelia cells), viability of cells appeared to be unaffected by fibre diameter. The ability of electrospun polymer scaffold to act as a conveyor for kidney cells makes them an ideal candidate within kidney tissue engineering; the non-woven path provides benefits over decellularised tissue by offering a high morphological control as well as providing superior mechanical properties with degradation over a tuneable time frame.

## Introduction

Chronic kidney disease (CKD) is a major worldwide health problem which attributes to 1.5% of deaths worldwide [1]. Current treatment involves dialysis and eventual transplantation; dialysis is a costly technique which constrains day to day life [2]. In 2016 there were 5275 people on the transplant list in the UK alone [3], this places huge stresses on health care providers as demand outstrips supply. Recent strategies to overcome these shortcomings in transplants are mainly focused on social policy with a new system of presumed consent being proposed [4]. Additional to this there are new avenues and endeavours in bioartificial kidney devices acting as miniaturised implantable dialysis devices which are showing great promise [5]. Furthermore, kidney tissue engineering is one avenue that could provide a better basis for treatment and understanding of CKD and has the unique potential to help meet the ever-growing demand for organs.

Initial success in kidney tissue engineering have shown potential as a testing platform for nephrotoxicity [6]. Current research has focused on simple microfluidic [7–11] and co-culture [12, 13] models representing structures such as the proximal tubules [7–9, 11] or glomerulus [10, 12, 13]; other approaches have included the use of embryonic or induced pluripotent cells cultured on tissue culture plastic creating immature kidney organoids [14–16]. A key element missing from these approaches is a 3D scaffold capable of providing mechanical strength, architectural cues and structure.

It is well documented that a 3D structure affects many different cell types [17–20] and could quite possibly be a crucial element of kidney tissue engineering. Decellularised tissue is currently the predominant vehicle for 3D kidney cell culture with impressive advances made [21–24]. This involves flushing detergent through renal tissue to wash out cells and DNA. This acellular extracellular matrix (ECM) is then recellularized and in some cases has been documented to produce rudimentary urine [25]. However, the decellularisation of tissue is not a simple process and the material left behind is often poorly characterised and mechanically weak [21, 26, 27], added to this the formidable task of recellularization [28] yielding enough uncertainty that other avenues should be pursued alongside.

Biomaterials such as polymer scaffolds have been investigated for kidney tissue engineering in a limited capacity, predominantly through investigation into hollow fibre bioreactors [29] but also as a conduit for renal segments [30]. Synthetic polymers, such as PLGA, have also been combined with natural ECM and have shown to enhance biocompatibility and reduce hydrophobicity of scaffolds compared to PLGA alone [31]. The paucity of research in polymer scaffold for kidney tissue engineering leave many avenues unexplored.

Electrospinning is a technique that has been utilised in many areas of tissue engineering to yield non-woven fibres resembling the ECM [32]. The technique produces consistent scaffold properties that can be precise when environmental parameters are finely controlled, including humidity, temperature, polymer concentrations, voltage–power, grounding and flow rate [33]. Morphologies of electrospun scaffolds can also be controlled and can affect how cells behave, larger fibres result in greater cell integration, nanofibers give a better representation of ECM and aligned fibres can induce linear orientation of cells [34–36]. Techniques such as cryogenic electrospinning have also demonstrated to increase the porosity of scaffolds allowing for greater cell integration [37]. In kidney tissue engineering electrospun collagen has been utilised to model the glomerular capillary wall, with evidence of cell–cell communication [13]. Electrospun polycaprolactone has previously been used for culturing epithelial and endothelial cell and shown to support cell life. Cells were seen to migrate into scaffolds, an outcome which is highly desirable for a multi-cell population required to create an organoid [38].

We have previously shown the ability of electrospun scaffolds to support a kidney cell line [39]. Here, for the first time to the authors knowledge, we study the growth of a multipopulation of rat primary kidney cells on poly(lactic acid) scaffolds of differing morphologies. With there being a distinct lack of research into polymer scaffold for kidney cells, here we show their intrinsic potential as a microenvironment that can maintain multiple cell phenotypic characteristics.

## Materials and methods

### Electrospinning

Poly(lactic acid) (PLA) (Goodfellow, UK) was spun at 3 different percentage weight by volume solutions in 1,1,1,3,3,3-hexafluoro-2-isopropanol (HFIP) (Manchester Organics, UK). Small fibres were created from a 10% w/v solution at + 17, − 2 kV with a flow rate of 0.5 ml/h at a needle to mandrel distance of 140 mm, needle bore of 0.4 mm. Medium fibres were created from an 18% w/v solution at + 15, − 4 kV with a flow rate of 4 ml/h at a needle to mandrel distance of 200 mm, needle bore of 0.8 mm. Large fibres were created from a 22% w/v solution at + 16, − 4 kV with a flow rate of 4 ml/h at a needle to mandrel distance of 23 mm, needle bore 0.8 mm. Cryogenic fibres were created using the same parameters but with a mandrel filled with dry ice (− 78.5 °C), topping up with dry ice each hour. The low temperature causes ice crystal deposition on the mandrel, increasing the porosity of scaffolds. After spinning the mandrel is freeze dried for 24 h, leaving behind a cryogenically modified scaffold. All fibres were spun at 250 rpm on to a mandrel covered in aluminium foil with 16 ml of solution used for each scaffold. Electrospun sheets were dried in a fume hood for 24 h to remove residual solvent and cut into 10 mm disks ready for cell seeding.

### Mechanical testing

Mechanical testing was performed using an Instron 3367 (Instron, UK) tensile testing machine. A gauge length of 20 mm and width of 5 mm was used for test pieces and thickness was measured using a digital micrometre. Samples were strained at 50% strain per minute with ultimate tensile strength and incremental Young’s modulus (between 0 and 5% strain in 1% intervals) calculated from an N ≥ 5 independent replicates, as previously described [40].

Estimated porosity was calculated by measuring the density of the scaffold and dividing it by the known density of PLA, as in the equation below:

$$ {\text{Porosity}} = \left( {1 - \frac{{{\text{Density}}\;{\text{of}}\;{\text{Scaffold}}}}{{{\text{Density }}\;{\text{of}}\;{\text{Polymer}}}}} \right) \times 100 $$

### Scanning electron microscopy

Fibres were coated using an Emscope SC500A splutter coater using gold–palladium (60:40). A Hitachi S4700 fuelled emission scanning electron microscope (SEM) (Hitachi) with a 5 kV accelerating voltage and a working distance of 12 mm was used to image scaffolds.

### Primary rat kidney isolation

Kidneys were taken from a 4-week-old female Sprague–Dawley rat and washed in Krebs–Ringer bicarbonate buffer supplemented with 1% antibiotic/antimycotic (anti/anti). The renal capsule and adjacent connective tissue were removed, and kidneys washed before placing in a falcon tube containing Krebs–Ringer and anti/anti. Kidneys were transferred to a cell culture hood and minced in a petri-dish using a scalpel. Minced tissue was incubated in collagenase from clostricium histolyticum (Sigma-Aldrich, UK) at a concentration of 0.625 mg/ml for 30 min at 37 °C, 2 kidneys per 12.5 ml. Following, the solution was filtered through a 70 µm cell strainer and neutralised with Dulbecco’s Modified Eagle’s Medium (DMEM) supplemented with 1% anti/anti and 10% foetal bovine serum (FBS). The solution was centrifuged at 500 g for 5 min and the supernatant discarded. Cells were resuspended in a 1:1 ratio of DMEM and keratinocyte serum-free media (KSFM) supplemented with 25 mg bovine pituitary extract and 2.5 µg epidermal growth factor, 5% FBS and 1% anti/anti. Cells were plated with 2 kidneys in 5 T175 flasks and cultured for 24 h before washing with PBS and replenishing media. Protocol adapted from He et al. [21] and Joraku et al. [41].

### Cell seeding

Scaffolds were sterilised in 2-propanol for 30 min before washing 3 times in phosphate buffered saline (PBS). Trypsin–EDTA 0.05% was used to lift cells from culture flasks, neutralised in cell culture media and centrifuged at 500 g for 5 min. In 12 well suspension plates, 400,000 cells (p1) were seeded to each scaffold in 50 µl media and left to attach for an hour before adding an additional 400 µl media. Cultures were incubated at 37 °C in 5% CO_2_ for 3 and 7 days and media was changed every 2 days.

### Cell viability

A CellTitre-Blue^®^ assay (Promega, UK) was used to evaluate cell viability. Scaffolds were placed in a new 48 well suspension plate and washed 3 times in PBS, 480 µl of stock solution (5:1, media/assay) was added to each scaffold and incubated for 2 h. Fluorescence was read using a microplate reader (Modulus II 9300-062, Turner Biosystems) at Ex 520 nm Em 580–640 nm, N = 4 independent replicates. A standard curve was used to estimate cell number.

### DNA quantification

Scaffolds were washed 3 times in PBS and freeze dried for 24 h. Each scaffold was incubated within an ultrasonic bath (6 litre Cavitek Digital, The Allendale Group, UK) in 300 µl of a 2.5 units/ml papain digest solution, 5 mM cysteine HCL and 5 mM EDTA in PBS (all reagents from Sigma Aldrich, UK) at 60 °C for 24 h with periodic sonication. Total DNA content of the samples was calculated using a Quant-iT™ PicoGreen^®^ assay kit (ThermoFisher, UK) as per the manufacturers’ instructions. Fluorescence was read using a microplate reader at Ex 490 nm Em 510–570 nm, N = 4 independent replicates.

### Immunohistochemistry imaging

Scaffold were washed 3 times in PBS and fixed in a 3.7% (v/v) solution of formalin and PBS for 10 min, followed by 3 additional washes. Permeabilization was performed using a 0.05% TWEEN in a 10 mM Tris and 1 mM MEDTA solution, scaffolds were incubated in 300 µl for 1 h before washing 3 times.

Scaffolds were dehydrated in 2-propanol solutions graduating from 30 to 100% for 10 min in each. Scaffolds were then left in a solution of 2-propanol and polyester wax (1:1) at 50 °C overnight. Next, scaffolds were placed in polyester wax for 3 h and then fresh wax overnight at 50 °C. Scaffold were halved and blocked for sectioning into 35 µm slices.

**Fig. 1:**
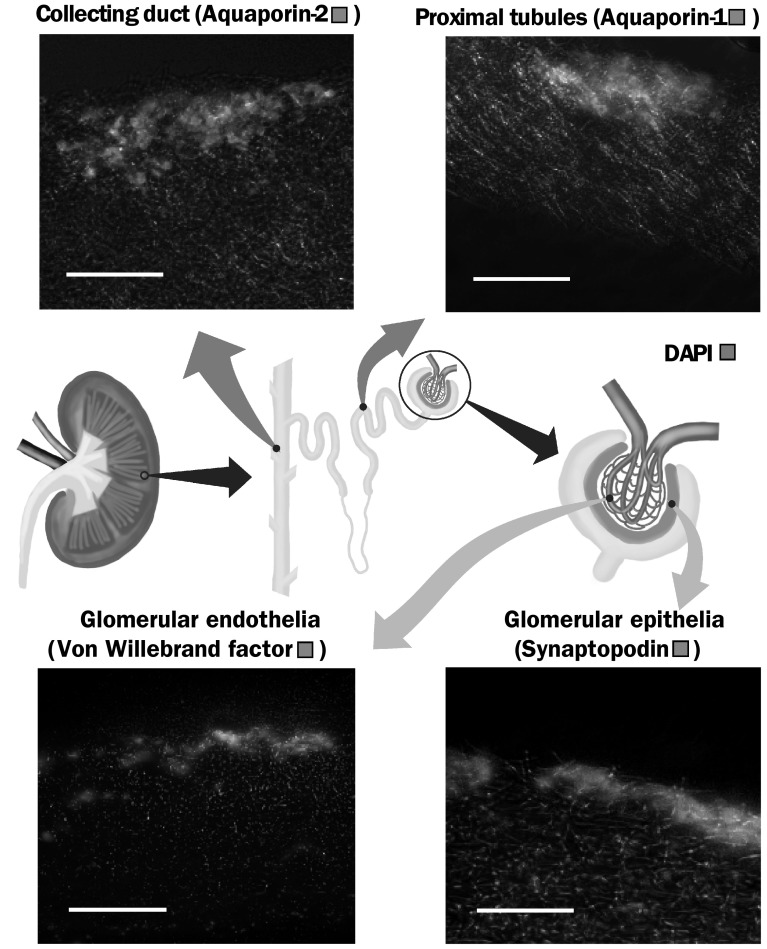
A diagram of the kidney showing the structure of kidney including the nephron and glomerulus, highlighting the location of key cell types, representative IHC images are taken from large scaffold at 7 days, scale bar is 100 µm

Cell were stained for DNA using 0.1 mg/ml 4′,6-diamidino-2-phenylindole (DAPI) (Sigma-Aldrich, UK) in PBS for 10 min. IHC was performed to establish the presence of key cell types including, primary antibodies aquaporin-1, aquaporin-2 and synaptopodin (Stratech, UK) were used at a 1 µg/ml dilution, von Willebrand factor (Abcam, UK) was used at 2 µg/ml (Fig. 1), and scaffold were incubated overnight in 10 µl, no-primary controls were used. Alexa Fluor 488 anti-rabbit IgG (ThermoFisher, UK) was used as a secondary antibody and left to incubate for 1 h before performing 3 washes, 5 min each. Imaging was done using a Zeiss Axio Imager fluorescence microscope.

### Statistical analysis

Data was tested using Levene’s test for equal variance before statistical analysis was performed in order to select appropriate tests. Electrospun fibre diameters were analysed using a one-way ANOVA with post hoc Games-Howel test. CellTitre blue and mechanical data was analysed using a one-way ANOVA with post hoc Tukey pairwise comparison; as data had a slight positive skew it was transformed by natural log giving a more normal distribution. Data on electrospun fibres is presented as mean ± standard deviation, all graphs are presented as mean ± 95% confidence interval, circles on graphs denote individual data points.

## Results

### Electrospun fibres

Non-woven fibres were created by electrospinning, it is clear that the variation in spinning parameters produced scaffolds of significantly different fibre diameters, F(3,87) = 2274, *p* = 0 (Fig. 1). Discrepancies were found between cryogenic and large fibre diameters, despite spinning using the same parameters, which is as a result of natural variation between spinning sessions and colder spinning environment. Cryogenic electrospinning produced a scaffold 5 times thicker than spinning with traditional methods, this rise in thickness dramatically increases the porosity from 82.5% for large fibres spun using the same parameters to 97%, but it does come at the cost of mechanical strength, Table 1.

**Table 1:** Mechanical properties and physical properties of PLA scaffolds

Average	Strain	Small	Medium	Cryogenic	Large
Fibre diameter, µm	-	0.88 ± 0.16	2.46 ± 0.43	3.71 ± 0.36	3.30 ± 0.17
Scaffold thickness, µm	-	193 ± 5.16	270 ± 8.63	1375 ± 160	218 ± 13.0
Porosity, %	-	86.9	82.8	97.3	82.5
Young’s modulus at % Strain, MPa	0–1%	2.84 ± 1.41	5.78 ± 1.51	0.57 ± 0.15	7.15 ± 1.97
	1–2%	6.53 ± 0.85	6.40 ± 1.49	0.72 ± 0.16	6.61 ± 0.81
	2–3%	5.34 ± 0.91	9.13 ± 1.12	1.13 ± 0.30	8.69 ± 1.48
	3–4%	4.76 ± 0.54	7.48 ± 0.94	1.31 ± 0.21	6.87 ± 1.62
	4–5%	2.84 ± 0.67	4.22 ± 0.62	0.81 ± 0.14	3.59 ± 1.13
	0–5%	5.05 ± 0.52	7.31 ± 0.84	1.01 ± 0.20	7.14 ± 1.04
Ultimate tensile strength, MPa		3.25 ± 0.21	4.25 ± 0.31	0.87 ± 0.17	4.02 ± 0.56

The Young’s modulus at 0–5% strain was analysed by one-way ANOVA, this analysis showed a significant difference between scaffolds F(3,21) = 103.32, *p* = 0, Table 1. Post hoc analysis using Tukey test showed that all scaffolds at 0–5% strain, except medium compared to large fibres, were significantly different to each other, *p* < 0.001. A similar trend follows with analysis of ultimate tensile strength, F(3,21) = 133.31, *p* = 0. Post-hoc analysis shows all scaffold are significantly different from each other except large and medium scaffolds, *p* < 0.05. Notably, randomly spun scaffolds reach a peak Young’s modulus at 2–3% strain whereas cryogenic spinning increases the percentage strain at which Young’s modulus is highest to 3–4% strain.

### Cell viability and DNA quantification

**Fig. 2:**
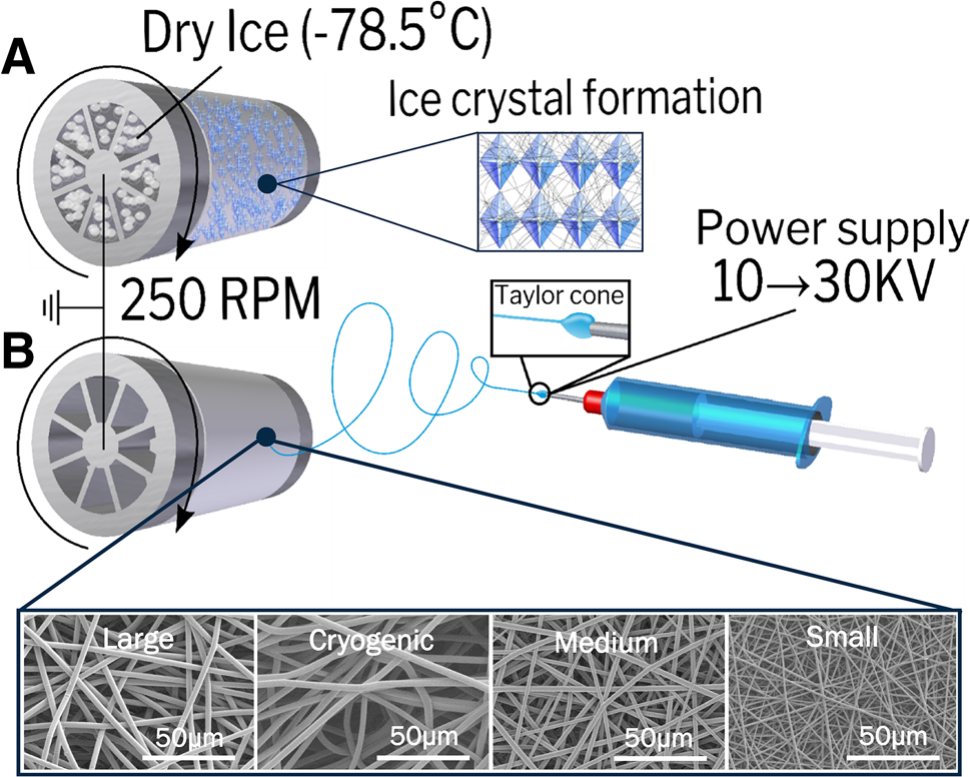
Scaffolds were fabricated by electrospinning, where a high voltage is applied to a polymer solution, forming a Taylor cone, this is then accelerated towards a ground source. Fibre architecture was determined by spinning parameters; **A** cryogenic fibres, using ice crystal formation as a template for fibre deposition and **B** random fibres onto a slowly rotating mandrel. SEM images below demonstrate the difference in fibre diameter of the scaffolds, which were spun using the same solvent and polymer but different electrospinning parameters and percentage weight solutions

CellTitre Blue^®^, which was used to determine cell viability, showed that cells did have a preference for a larger fibre diameter however these differences were not significant, the same applies to the number of viable cells from 3 to 7 days, F(7,24) = 2.05, *p* = 0.090. However, when compared to cells grown on tissue culture plastic there were significantly more cell present within the 12 well plate control F(9,30) = 18.23, *p* = 0, with post hoc analysis showing significantly more cells on tissue culture plastic than all scaffolds, Fig. 2.

**Fig. 3:**
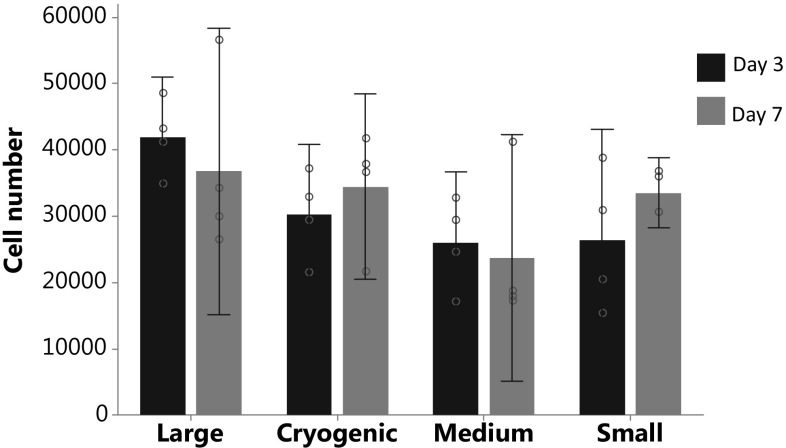
Cell number estimated from a standard curve, analysed using a CellTitre blue^®^ fluorescence assay. This demonstrates the ability of all scaffold architectures to support primary kidney cell life. No significant differences found in analysis using a one-way ANOVA F(7,24) = 2.05, *p* = 0.090. Data presented as mean ± 95% confidence intervals, circles show individual data points

Analysis of DNA quantification, determined by PicoGreen assay, using a one-way ANOVA showed a significant difference between groups, F(7,23) = 4.79, *p* = 0.002. Post-hoc Tukey test highlighted that cryogenic scaffolds at day 3 was significantly different from all groups except cryogenic at day 7 and medium at day 3, no other significant differences were seen. DNA quantification validates the fact that the number of cells is does not increase from 3 to 7 days, Fig. 3.

### Immunohistochemistry

**Fig. 4:**
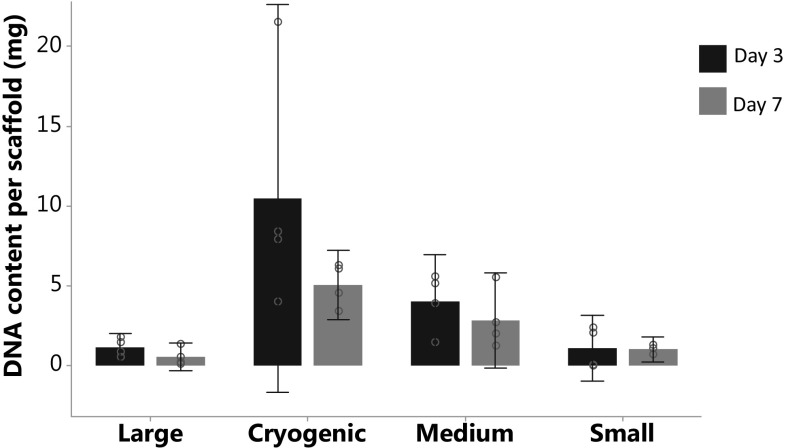
DNA quantity per scaffold at 3 and 7 days, assessed by PicoGreen assay. This confirms the ability of all scaffold architectures to support primary kidney cell life. Analysis using a one-way ANOVA showed significant differences F(7,23) = 4.79, *p* = 0.002, post hoc Tukey analysis shows that was in regards to cryogenic scaffolds. Data presented as mean ± 95% confidence intervals, circles denote individual data points

Immunohistochemistry (IHC) showed the presence of key signatures of several cell types: aquaporin 1 and 2 showed the presence of tubular cells, synaptopodin highlights glomerular epithelial cells and von Willebrand factor indicates glomerular endothelial cells. As seen in Fig. 4, these key markers are seen on all scaffold types, demonstrating the presence of a multi-population of cells. The sectioned scaffold show that cells were seen throughout cryogenic scaffolds, but less cell penetration was present on all other fibres types.

## Discussion

Emphasis so far in kidney tissue engineering has been on 2D cell culture, which has shown considerable progression in recent years [15, 42, 43]. However, cells within the Kidney do not exist within the two dimensional axis of cell culture plastic, with greater amounts of research underlining the importance of a 3D structure [17, 44]. 3D kidney tissue engineering has focused on the use of decellularised tissue with promising results [25, 45]; this method is not without its shortcomings, with issues surrounding decontamination [46] and a lack of a standardized approach to decellularisation leading to a variation between scaffolds [21, 26, 27]. Decellularised tissue brings favourable physical and chemical characteristics to help support cells and control physiology [47], but as cell produce their own ECM a synthetic scaffold can provide an excellent foundation to build upon [48, 49]. Polymer scaffolds offer many benefits over decellularised tissue as their manufacture can be highly controlled, as well as providing superior mechanical properties with degradation over a tuneable time frame [31, 33, 50].

Our electrospun fibres demonstrate the control with which polymer scaffold are fabricated having a standard deviation in fibre diameter and Young’s modulus (0–5% strain) of less than 20% in all cases, Table 1. The increase in porosity of 97.3% gained from cryogenic electrospinning, demonstrated in the dramatic increase in thickness of the scaffold spun under the same conditions as large fibres, comes at the cost of mechanical strength; fibres had an UTS 5 times lower despite spinning under the same parameters, this follows a similar trend to previous works [37, 51]. The variation seen in scaffold thickness may be influenced by a decrease in humidity due to the low temperature as humidity is known to effect the diameter of electrospun fibres [52], the low temperature of the mandrel may also reduce the ambient temperature increasing the viscosity of the polymer solution [53]. Interestingly, Young’s modulus peaks in normally spun scaffolds at 2–3% strain but at 3–4% for cryogenically spun fibres this is probably due the nature of the scaffold and looser packing in cryogenic fibres allowing for more strain in the scaffold before all fibres are in tension. Cryogenic scaffold can be controlled further thought monitoring the humidity of spinning conditions [54].

There is great scope for the use of polymer scaffolds within kidney tissue engineering. Our work follows on from the limited published research into the use of kidney cells with electrospun scaffolds [13, 30, 55]. We have previously shown that electrospun PCL can be used to grow a kidney cells line, and highlighted the effects of morphological differences in scaffolds [39]. Here we have gone further, using RPK cells and demonstrating the survival of key cell types of this multi-cell population, Fig. 4. Although, our results regarding fibre diameter and its influence on cell behaviour are not conclusive, it has been shown to have major implication in previous studies [39, 56–58]. There is some disparity between our data for CellTitre blue and DNA quantification, this is possibly as a result of the much greater porosity gained from cryogenic electrospinning where unattached cells become trapped within the scaffold. It does however show that viable cells, shown through CellTitre blue, are surviving over time as DNA content remained constant.

An important factor to consider here is scaffold chemistry, we have demonstrated that PLA facilitates primary cell survival but it is not optimised for kidney cells. Survival on polymer scaffold could be vastly improved with optimisation through such techniques as microarray [59–61] allowing for analysis of cell interaction with many polymer types in a high-throughput manner. Other popular optimisation techniques include integrating ECM components with polymer scaffold to produce a hybrid deriving benefits from both, this method has shown to improve the mechanical properties of the scaffold whilst increasing cell interaction [31]. Further novel techniques have used a sacrificial cell layer to produce an ECM layer on top of the polymer scaffold before decellularisation, maintaining the initial mechanical characteristics [49]. Some of these methods may bring about a greater interaction between cells and scaffold and could provide more conclusive evidence on the type of architecture that is most favourable to kidney cells.

**Fig. 5:**
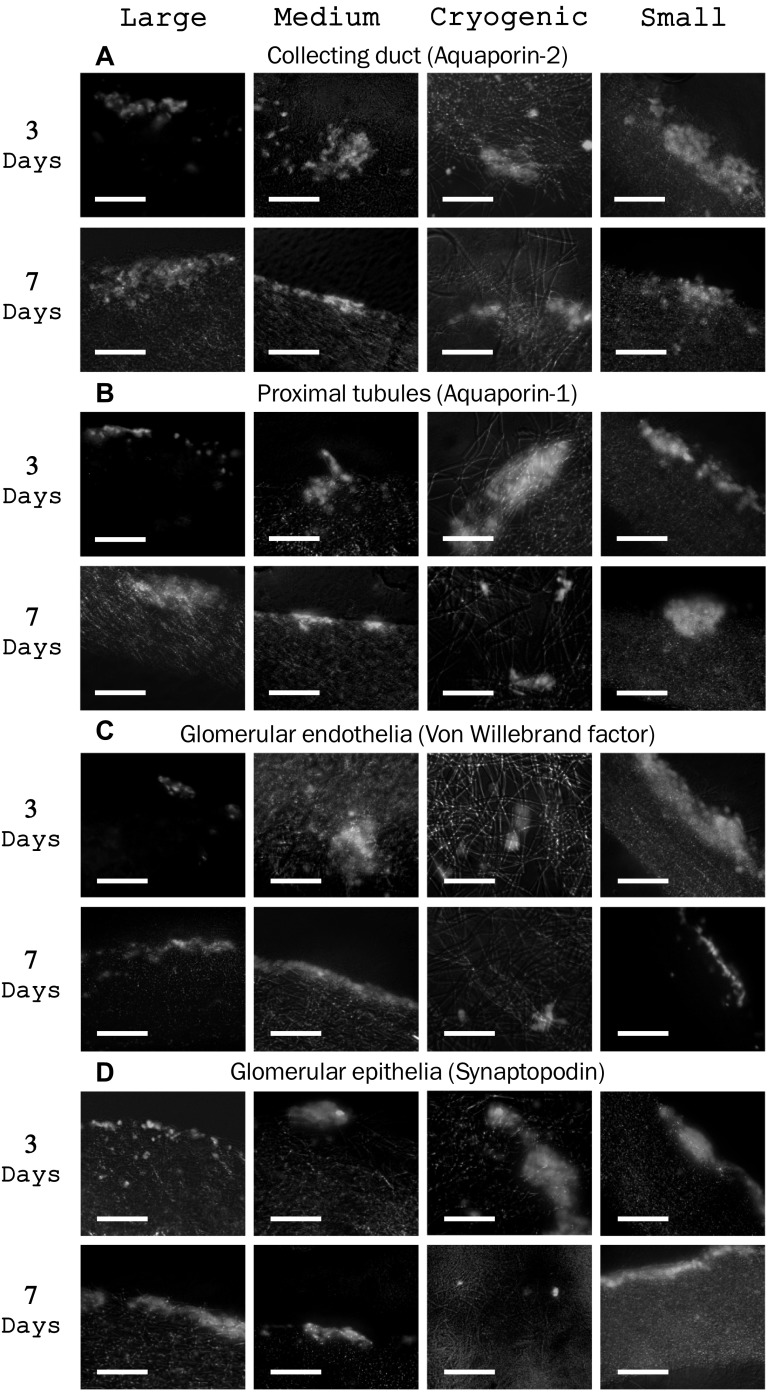
Fluorescence images showing DAPI and IHC, used to show the presence of key functional marker of several cell types: **A**–**D** aquaporin-2, aquaporin-1 indicate the presence of tubular cells, von Willebrand factor indicates glomerular endothelial cells and synaptopodin indicated the glomerular epithelia, scale bar is 100 μm

Fluorescence IHC images (Fig. 5) clearly shows the presence of key markers of essential kidney cells (Fig. 1). The marker which identified key cells were: aquaporin-1, aquaporin 2, Synaptopodin and von Willebrand factor, between them these highlight the cells which make up the proximal tubules, collecting duct, glomerular epithelia and glomerular endothelia, Fig. 1. The proximal tubules lead from the loop of Henle and are responsible for the reuptake of filtrate, they consist of epithelial cells with microvilli to increase surface area [7]. The collecting duct is the last stage of filtration where the filtrate is reabsorbed and collected. The glomerular epithelia are more commonly referred to as podocytes and form part of the glomerulus, playing a key role in blood filtration through slits which block the passage of larger molecules [62]. The glomerular endothelium are another key component of the glomerular characterised by fenestrations which are essential for filtration [63]. In order for any kidney tissue engineering scaffold intended to host a multi-cell population to be considered successful it is essential to show the survival of these key components; as can clearly be seen on our scaffolds cells were displaying initial integration with the 3D structure. The sectioned scaffolds did not fully integrate through the full depth in traditionally spun scaffolds, this is a common problem with electrospun fibres, reported in other works [64, 65]. Pore size of the scaffold is the predominant factor which limits cell infiltration, naturally the larger the fibre the larger the pore size; methods such as co-spinning of micro and nanofibres has been used to increase this pore space [33, 66], as well as using a sacrificial dissolvable polymer in a similar co-spinning manner [67]. Here we demonstrate the use of cryogenic spinning [51, 54], utilising ice crystals as a template the pore size is dramatically increased. Although our results on the most favourable morphology were inconclusive, we have demonstrated the greater cell integration cryogenic scaffold can bring, and have shown the potential of electrospun PLA as a scaffold for rat primary kidney cells and its capacity to maintain the multi-population of cells.

We did not see organisation of cells into kidney like structures and more research is needed over a longer time frame to assess for elements of self-organisation. The ability of embryonic and induced pluripotent stem cells to self-organise is well documented [6, 68], and these cells of greater physiological and clinical relevance are the next logical step for polymer scaffolds in kidney tissue engineering. Our view is that polymer scaffolds have the ability to act as a conveyor for kidney cells, allowing kidney cells to self-organise before implantation where a full organ can mature. We feel this is a reasonable alternative to the implantation of decellularised organs, and has previously been highlighted as a potential avenue [69].

We have demonstrated here the variation of architectures that can be created from a single polymer and solvent solution by electrospinning. This is just one polymer in many that could be used in kidney tissue engineering. Electrospun polymer scaffolds have the ability to create a range of different architectures and should be considered for further investigation in kidney tissue engineering due to their: ability to host a multi-population of cells, biocompatibility, reproducibility, good mechanical properties and 3D structure. This is a new non-woven path within kidney tissue engineering and one that should be explored further.
